# Community Pharmacists’ Knowledge, Attitude, and Practice in Providing Self-Care Recommendations for the Management of Premenstrual Syndrome

**DOI:** 10.3390/medicina56040181

**Published:** 2020-04-15

**Authors:** Muhammad Taufik Suaidi, Poh Kuan Wong, Nurul Ain Mohd Tahir, Eng Wee Chua

**Affiliations:** Faculty of Pharmacy, Universiti Kebangsaan Malaysia, Jalan Raja Muda Abdul Aziz, Kuala Lumpur 50300, Malaysia; taufikmikhail96@gmail.com (M.T.S.); pkwong96@hotmail.com (P.K.W.); nurulainmt@ukm.edu.my (N.A.M.T.)

**Keywords:** premenstrual syndrome, self-care, community pharmacists, over-the-counter products, symptomatic therapy

## Abstract

*Background and objectives*: Premenstrual syndrome (PMS) comprises a variety of physical and emotional symptoms that affect women of reproductive age. The distress caused by PMS often leads to self-medication, and many over-the-counter or non-prescription products are available for relieving PMS symptoms. The choice of a suitable product should be based on advice from a health professional, such as a community pharmacist. Hence, we assessed the knowledge, attitude, and practice of Malaysian community pharmacists in providing self-care recommendations for the management of PMS. *Materials and Methods*: A cross-sectional survey was carried out in Kuala Lumpur, Malaysia from September to November 2018 using a self-administered questionnaire. The respondents were community pharmacists working in Kuala Lumpur and were chosen from a list of Type A license holders in the city. *Results*: We achieved a response rate of 79% and included 181 questionnaires in the final analysis. Of the 181 respondents, most of them (76.8%; n = 139) had medium to good levels of knowledge of PMS. Likewise, most of the respondents (78.5%; n = 142) had positive attitudes toward their role in PMS management. Having taken courses on managing minor illnesses in women substantially enhanced their levels of knowledge of (*p* = 0.002), but not their attitude towards, PMS management. Among the PMS-relieving products, the most commonly recommended products were ibuprofen (79%; n = 143), mefenamic acid (74.5%; n = 135), and naproxen (66.9%; n = 121), which are well known for their anti-inflammatory effect. This suggests that the respondents based their product choice on sound evidence. *Conclusions*: Community pharmacists can play an important role in the management of PMS. In future work, a larger sample can be assembled to obtain more insight into the readiness of community pharmacists to help women in self-managing PMS and establish a specialized service to this end.

## 1. Introduction

Premenstrual syndrome (PMS) is a recurrent luteal phase condition characterized by physical, psychological, and behavioral changes that impair daily activities [1]. Common psychological symptoms include changes in mood, irritation, and depression, and frequently reported physical symptoms include breast tenderness, bloating, and headache [2].

A variety of treatments are used to relieve PMS symptoms. For women with mild symptoms, supportive counseling, education, increased exercise, and eating a healthy diet are effective counter-strategies [3,4], but pharmacological treatment is needed for more severe PMS. For instance, non-steroidal anti-inflammatory drugs (NSAIDs), gonadotropin-releasing hormone agonists, and oral contraceptives are used to temporarily eliminate PMS symptoms [5,6]. Natural products and health supplements, such as saffron, chasteberry, and calcium, are slow-acting but may be helpful nonetheless in alleviating PMS [7,8].

A severe form of PMS, premenstrual dysphoric disorder (PMDD), causes depressive symptoms and warrants the use of antidepressants such as fluoxetine and sertraline, which inhibit the neuronal reuptake of serotonin [9]. Women with PMDD typically suffer substantial functional impairment, and prompt diagnosis is necessary to inform treatment decisions [10].

However, some women may find neither the long-term use of prescription medications, which could cause substantial side effects, nor lifestyle modifications sufficient to manage the symptoms. The need for safe and effective over-the-counter (OTC) products have prompted many women to consider non-prescription products for PMS [11]. Many non-prescription products are useful for alleviating PMS symptoms.

However, the clinical evidence for the use of OTC products in managing PMS is weak. Their efficacy has been established mostly based on anecdotes [8]. Because OTC products have shown some efficacy in the treatment of PMS, this has led to self- medication among women with PMS [11]. In Malaysia, a variety of OTC products are available for managing PMS, and Malaysian women prefer OTC products over prescription medications [12].

Community pharmacists are gatekeepers ensuring the proper use of OTC products to manage minor illnesses. In the management of PMS, they give advice on use of the OTC products in relieving mild PMS symptoms and refer women with severe PMS, or PMDD, to gynecologists [13]. However, a prior study has shown that community pharmacists have not been sufficiently involved in the management of PMS. The study also showed that community pharmacists did not ask enough questions to make appropriate recommendations and that their recommendations were not evidence-based [11]. To ensure that the OTC products recommended to customers with a PMS-related complaint are effective, community pharmacists should have adequate knowledge and a positive attitude about the management of PMS. Hence, in this study, we assessed the knowledge, attitudes, and practices of community pharmacists in providing self-care recommendations for the management of PMS.

## 2. Materials and Methods

### 2.1. Study Design

This was a cross-sectional survey conducted among the pharmacists working in Kuala Lumpur, Malaysia. An information sheet was given to the respondents to give them an overview of the study. On agreeing to participate in the study, the respondents signed a written informed consent form before they received a self-administered questionnaire. We started the survey on 15 September 2018 and completed it on 30 November 2018.

### 2.2. Subject Sampling Mechanism

We recruited the participants for this study through convenience sampling and selected them based on a list of Type A (Poison) License holders in Kuala Lumpur, which was available from the website of the Pharmaceutical Services Division, Ministry of Health Malaysia. The list included community pharmacists who worked in the eleven parliamentary regions in Kuala Lumpur, namely Titiwangsa, Setiawangsa, Seputeh, Bandar Tun Razak, Wangsa Maju, Lembah Pantai, Bukit Bintang, Segambut, Batu, Cheras, and Kepong. At the time of subject selection, there were 563 registered community pharmacists in Kuala Lumpur. We used the Krejcie and Morgan (1970) formula [14] to calculate the sample size:

(1) $$s=x^{2}NP\left(1-P\right)/d^{2}\left(N-1\right)+x^{2}P(1-P)$$

where *s* is the required sample size, *N* is the population size (563), *x^2^* is the table value for chi-square for 1 degree of freedom at the desired confidence interval (3.841), *P* is the population proportion (assuming 0.5 as this would provide the maximum sample size), and *d* is the degree of accuracy expressed as a proportion (0.05). The target sample size was calculated at 229.

### 2.3. Sampling Criteria

The respondents for this study must meet the following inclusion criteria: (1) they must be community pharmacists who were fully registered with the Pharmacy Board of Malaysia and (2) they must be able to read and understand English. Some respondents met the inclusion criteria but were not allowed by their employer to participate in the study. Also, we excluded some incomplete questionnaires from our analysis, and this reduced the final sample size.

### 2.4. Study Instrument

We wrote the questionnaire based on prior surveys [11,15,16,17,18,19]. We divided it into five sections, which contained 49 questions in total (see supplementary materials online).

- Section A (Sociodemographic Information)
- Section B (Knowledge of Premenstrual Syndrome and Its Management)
- Section C (Attitude of Community Pharmacists toward Their Role in Providing Recommendations for Managing Premenstrual Syndrome)
- Section D (Pharmacological Recommendations)
- Section E (Non-Pharmacological Measures)

We used 14 questions to assess the respondents’ knowledge of the definition, the symptoms, and the management of PMS. Each question had three options, namely ‘true’, ‘false’, and ‘I do not know’. A score of 1 was given to a correct answer, and no score was given to an incorrect answer or when the respondents chose ‘I do not know’. Bloom’s cut-offs are commonly used to assess knowledge and attitude levels among survey respondents [20,21]. We used the Bloom’s cut-offs, i.e., 80.0%–100.0% (12–14 points), 60.0%–79.0% (9–11 points), and ≤59.0% (<9 points), to classify the respondents as having high, medium, or low levels of knowledge of PMS.

We used 12 statements to gauge the respondents’ attitude towards their role in PMS management. We gauged how much they agreed or disagreed with each statement using a five-point Likert scale: 1-strongly disagree, 2-disagree, 3-neutral, 4-agree, 5-strongly agree. We then rated their attitudes using the Bloom’s cut-offs, i.e., positive (80–100%, or 48–60 points), neutral (60–79%, or 36–47 points), or negative (<60%, or <36 scores).

### 2.5. Pilot Test

We carried out a pilot survey among 20 community pharmacists from Kuala Lumpur. The purpose was to assess the internal consistency and the clarity of the questionnaire. We then made several minor changes to the questionnaire based on the findings of the pilot survey. We did not include the data we obtained from the pilot survey in the final analysis.

### 2.6. Ethical Approval

This project was approved by the Research Ethics Committee of Universiti Kebangsaan Malaysia on 29th June 2018 (UKM PPI/111/8/JEP-2018-356).

### 2.7. Data Analysis

We analysed the data using the Statistical Package for the Social Sciences (SPSS) version 25.0 (SPSS Inc., IBM, Chicago, IL, USA). We presented the data in medians alongside values for the interquartile range, and frequencies and percentages. We performed Mann–Whitney U tests to compare the knowledge scores and the attitude scores between the respondents, grouped based on their demographic characteristics, namely gender, employment status as a community pharmacist, years as a fully registered pharmacist, and years as a community pharmacist. In gauging the probability of making a type 1 error, we set the confidence level to 95% and used a *p*-value of <0.05 to mark statistical significance.

## 3. Results

### 3.1. Demographic Characteristics

We distributed a total of 229 questionnaires by hand to the community pharmacists working in Kuala Lumpur. However, only 181 agreed to participate in the study and completed the questionnaire, giving a response rate of 79%.

Of the 181 respondents, 98 (54.1%) were male and 83 (45.9%) were female (Table 1). Most of the respondents were between 21 and 40 years old (82.3%; n = 149); the rest were aged between 41 and 60 (17.7%; n = 32). The majority of the respondents were Chinese (82.9%); 10.5% were Malay; 5.5% were Indian; and 1.1% were from other ethnic groups. Most of the respondents (87.3%) worked as a full-time community pharmacist, and 12.7% were locums.

Most of the respondents held a bachelor’s degree of pharmacy (90.1%; n = 163); 8.8% (n = 16) had a master’s degree; and only one respondent (0.6%) had a PhD degree. Among the respondents, 71.3% graduated from a local university, and 28.7% graduated from an overseas university. Many of the respondents were fairly new to the profession, with 51.4% having worked as a fully registered pharmacist for only 1–5 years; 19.3% had 6–10 years of post-registration experience; 29.2% had worked in the profession for more than 10 years. Many of the respondents started working in the community setting once they had become a fully registered pharmacist. When asked about their work experience as a community pharmacist, 65.2% of the respondents replied that they had 1–5 years of experience; 14.4% had worked at a community pharmacy for 6–10 years; 20.4% had more than 10 years of experience as a community pharmacist.

We also asked the respondents whether they had relevant training in PMS management, as this could influence their knowledge of PMS. Most of the respondents (63.5%; n = 115) replied that during their undergraduate years at pharmacy school, they had taken courses on managing minor illnesses in women. However, many of the respondents felt that the courses were not effective. When asked about ‘where did they learn the most about managing PMS’, nearly half the respondents (47.5%; n = 86) chose ‘practice experience’; 41.4% (n = 75) thought that the undergraduate training was adequate; 11.0% (n = 20) chose continuing education. The respondents also had varied levels of experience in helping women with PMS. A large proportion of the respondents (45.9%, n = 83) replied that they only had to tackle PMS-related complaints monthly; 37.6% (n = 68) weekly, 11.0% (n = 20) daily, and 5.5% (n = 10) yearly.

### 3.2. The Respondents’ Knowledge of PMS and Its Management

We used 14 statements to assess the respondents’ knowledge of PMS and its management (Table 2). Most of the respondents chose the correct answer for the definition of PMS (89.0%; n = 161) and recognized the affective (92.3%, n = 167) and the somatic (93.4%; n = 169) symptoms of PMS. Many of the respondents also knew that PMS could harm work productivity (95.0%, n = 172) and impair sleep (97.2%; n = 176) among women suffering from the condition. Similarly, most of the respondents (97.2%; n = 176) knew that ibuprofen alleviates PMS symptoms; however, spironolactone was much lesser known among them (48.6%, n = 88). Interestingly, many of the respondents (68.5%, n = 124) were confused about the use of sertraline in PMS management. This suggests that they did not know the difference between PMS and PMDD, which is relieved by sertraline. Some of the respondents were not well versed in the non-pharmacological treatment of PMS; they were unaware of the benefit of regular exercise to PMS (21.0%, n = 38) or the augmenting effect of caffeine on the risk of PMS (61.9%, n = 112).

Based on the Bloom’s cut-offs, we found that most of the respondents (60.2%; n = 109) had a medium level of knowledge of PMS (Table 3). Then, we examined the factors that could influence the respondents’ knowledge of PMS and its management (Table 4). Mann–Whitney U tests revealed that the respondents who had taken courses on minor illnesses in women had significantly higher levels of knowledge of PMS (U = 2745, *p* = 0.002). No differences were found for the other demographic characteristics, namely gender (U = 3831.5, *p* = 0.484), employment status as a community pharmacist (U = 1459.5, *p* = 0.122), experience (years) as a fully registered pharmacist (U = 3148, *p* = 0.440), and experience (years) as a community pharmacist (U = 2471, *p* = 0.491).

### 3.3. The Respondents’ Attitudes toward Their Role in PMS Management

Table 5 shows the respondents’ attitudes toward their role in PMS management. Overall, the respondents believed that knowing how to manage PMS symptoms is important. Most of the respondents (90.6%; n = 164) agreed that ‘pharmacists should know which medicine is used to relieve PMS’; 87.3% (n = 158) agreed that ‘pharmacists should know the non-medical way to ease PMS’. When asked whether they should allocate ample time for ‘providing self-care recommendations to patients with PMS’, 81.3% (n = 147) thought that this was an essential part of their daily practice. More importantly, 82.8% (n = 150) agreed that they should help patients with choosing a suitable drug product for PMS, and 87.9% (n = 159) thought that product selection should be based on the scientific evidence.

We also used several statements to gauge the respondents’ willingness to provide drug- or product-related information to their customers. Here, 96.6% (n = 175) thought that they should provide patients with enough information on ‘the use of medicine’, and 95.6% (n = 173) agreed that this should include the instructions for the dosing schedule and the drug administration. Furthermore, 90.6% (n = 164) agreed that they should tell patients about the potential side effects of a drug product. A similar number of respondents (90.6%; n = 164) thought that they should always check whether patients understand their instructions.

Then, we also assessed the respondents’ readiness to respond to PMS-related inquiries. Here, 93.4% (n = 169) felt that they should be ready to respond to requests from customers for a PMS-relieving product. However, considerably fewer respondents (66.9%; n = 121) agreed that ‘patients regularly accept their recommendations for PMS-relieving products’. Nevertheless, many of the respondents (91.1%; n = 165) remained willing to provide recommendations for managing PMS when given the opportunity.

Overall, many of the respondents (78.5%; n = 142) had positive attitudes toward their role in PMS management (Table 6). We also examined the factors that could influence the respondents’ attitude towards their involvement in PMS management. Table 7 shows that there was no significant relation between the respondents’ demographic profiles and how they viewed their role in PMS management. We examined the following demographic characteristics, namely gender (U = 3830, *p* = 0.486), position as community pharmacist (U = 1681, *p* = 0.561), years as a fully registered pharmacist (U = 3340, *p* = 0.871), years as community pharmacist (U = 2242.5, *p* = 0.137) and having taken courses on managing minor illnesses in women at pharmacy school (U = 3311.5, *p* = 0.153).

### 3.4. The Respondents’ Pharmacological Recommendations for Relieving PMS Symptoms

Table 8 lists the PMS symptoms often or always reported to the respondents by their customers. The most frequently reported symptom was headache (64.6%; n = 117), followed by irritability (62.4%; n = 113), abdominal bloating (60.3%; n = 109), anger (40.3%; n = 73), breast pain (35.9%; n = 65), anxiety (25.4%; n = 46), swelling of extremities (24.8%; n = 45), confusion (9.4%; n = 17), depression (17.1%; n = 31), and social withdrawal (13.8%; n = 25).

Table 9 shows the products that were recommended by the respondents to their customers for the symptomatic treatment of PMS. Ibuprofen (79.0%; n = 143), mefenamic acid (74.5%; n = 135), and naproxen (66.9%; n = 121) were most preferred by the respondents. Equally important in PMS treatment are health supplements and natural products, of which the most favored product was evening primrose oil (62.9%, n = 114), followed by soy (41.5%; n = 75), magnesium (33.7%; n = 61), vitamin E (32.6%; n = 59), calcium (27.6%; n = 50), vitamin B6 (16.6%; n = 30), St. John’s Wort (11.1%; n = 20), chasteberry (6.7%; n = 12), and saffron (5.5%; n = 10).

Table 10 lists the products that were used by women with PMS to manage their symptoms. The products that were frequently requested by them were mefenamic acid (87.7%; n = 148), ibuprofen, (79.0%; n = 132), and naproxen (72.9%; n = 132). Among the health supplements and natural products, the most popular products were evening primrose oil (19.9%; n = 36) and soy (19.9%; n = 36), followed by magnesium (17.1%; n = 31), calcium (16.6%; n = 30), vitamin E (13.2%; n = 24), vitamin B6 (9.4%; n = 17), St. John’s Wort (4.4%; n = 8), saffron (3.3%; n = 6), and chasteberry (3.3%; n = 6). We noted similar trends for the drugs or health supplements or natural products most preferred by the respondents, suggesting that customer requests influenced heavily the respondents’ choice of a product for tackling PMS symptoms.

### 3.5. The Respondents’ Pattern of Recommendations for Non-Pharmacological Management of PMS

Table 11 shows the pattern of the respondents’ recommendations for the non-pharmacological management of PMS. Most of the respondents (75.7%; n = 137) felt that ‘eating a healthy and balanced diet’ was an effective strategy for managing PMS, followed by ‘rest and sleep more’ (74.6%; n = 135), ‘exercise’ (69.1%; n = 125), ‘consult the doctor if the symptoms persist’ (69.1%; n = 125), ‘avoid caffeine and alcohol’ (68.0%; n = 123), ‘avoid stressful situations’ (56.9%; n = 103), and ‘use an antipyretic if there is a fever’ (47.0%; n = 85).

We also asked the respondents why they would encourage their customers to self-manage PMS symptoms (Table 12). Many of the respondents (81.8%; n = 148) replied that ‘ease of availability and convenience’ was a primary reason. This was followed by ‘cost-saving’ (56.4%; n = 102), ‘time-saving’ (31.5%; n = 57), ‘illness too trivial for consultation’ (30.9%; n = 56), ‘privacy’ (24.3%; n = 44), ‘to avoid crowd at outpatient department’ (7.7%; n = 14), and ‘a lack of trust in prescribing doctors’ (4.4%; n = 8). We also allowed the respondents to write down reasons that were not listed in the questionnaire. Some respondents gave us the following reasons: ‘painkillers are effective at treating PMS’, ‘PMS symptoms are readily manageable’, and ‘customers are knowledgeable enough to self-manage PMS symptoms’.

We then asked the respondents why they would discourage their customers from self-managing PMS symptoms (Table 13). Some of the respondents (76.8%; n = 139) chose ‘risk of using a wrong medication’ as the main reason. This was followed by ‘risk of adverse effects’ (69.6%; n = 126), ‘risk of disease misdiagnosis’ (69.1%; n = 125), ‘risk of drug interactions’ (58.0%; n = 105), and ‘risk of drug abuse and dependence’ (51.9%; n = 94).

## 4. Discussion

Community pharmacists can be an important source of self-care recommendations for the management of PMS, which is a distressing condition affecting many women. A prior survey suggested that community pharmacists should expand their role in the management of PMS and base their recommendations for PMS-relieving products on sound clinical evidence [11]. In this study, we assessed the knowledge, attitudes, and practices of Malaysian community pharmacists in providing self-care recommendations for the management of PMS.

### 4.1. The Respondents’ Knowledge of PMS and Its Management

Overall, many of the respondents had a medium level of knowledge of PMS and its management. Several prior surveys that were conducted among medical students, school children, and adolescent females reported variable findings [2,22,23]. In some of the studies, the participants were found to have limited understanding of PMS, i.e., its incidence, causes, and treatment. The other studies reported similar findings to ours. In a Karachi study that involved medical students, the participants were found to have good understanding of PMS [24]. In contrast, the women in three metropolises of Pakistan were found to have limited knowledge of PMS [25]. The difference highlights how one’s educational background can shape their knowledge of PMS. Community pharmacists in Malaysia are knowledgeable about PMS. This should enable them to assess and counsel women with PMS and set therapeutic goals to achieve better outcomes of PMS treatment [26].

Understanding certain aspects of PMS is especially key to its management. Many of the respondents knew that PMS could adversely affect quality of life. In a cross-sectional study conducted among female university students in Karachi, 60.4% of the participants reported that PMS affected their normal routine, 35.4% reported missing school or work, and 40.2% reported missing social events because of PMS [24].

The respondents knew the drugs commonly used for treating PMS. Many of them correctly chose ibuprofen, but not spironolactone, as a drug for relieving PMS. Physical symptoms such as tenderness in the breast, cramps, bloating, abdominal pain, and headache are typically treated with symptom-specific drugs. NSAIDs, such as ibuprofen, are most useful for relieving somatic pain and cramps [27]. The use of spironolactone, a potassium-sparing aldosterone-receptor antagonist, to reduce water retention is not well-founded [28]. This may explain why the drug was lesser known than ibuprofen among the respondents. Likewise, 68.5% of the respondents did not know or were unsure about the use of sertraline in PMS management. This suggests that many community pharmacists may not be able to differentiate between PMS and PMDD—the latter being a severe variant of the former and requiring different treatment. We did not assess the respondents’ knowledge of the other differences between PMS and PMDD, and this constitutes a limitation of our study.

Although many of the respondents felt that the training they previously received in managing minor illnesses in women was not effective, our study showed that having taken such courses significantly enhanced their levels of knowledge of PMS. This is in keeping with a study that assessed pharmacy students’ grasp of pharmacovigilance. The study found that prior (formal) training significantly influenced the students’ knowledge of pharmacovigilance [29]. We suggest that pharmacy schools should design and implement courses on minor illnesses in women to prepare the graduates for the management of these conditions at community pharmacies.

### 4.2. The Respondents’ Attitudes toward PMS Management

Community pharmacists are typically regarded as the guardians of public health, as they provide essential advice on the use of medications [30]. In our survey, most of the respondents had positive attitudes toward their role in PMS management. For instance, they believed that pharmacists should know the drug products or the non-pharmacological measures that relieve PMS. They also felt that they should help their clients in choosing a suitable self-care medication for PMS. Our findings coincide with other studies, in which pharmacists were deemed to play an important role in making product recommendations for managing PMS [11].

We also found that many of the respondents were ready to advise their customers on medication use, which included the dosing schedule, the method of drug administration, and the potential side effects. They agreed that they should always check whether their patients understand the instructions they provide. This is in keeping with the result of a survey conducted in Selangor, Malaysia, where 56.1% of the respondents agreed that pharmacists gave them enough drug-related information and encouraged them to ask questions before they bought a medication [31]. According to the Code of Ethics published by the Royal Pharmaceutical Society of Great Britain, pharmacists must ensure that ‘a patient receives sufficient information and advice to enable the safe and effective use of medicines’ [32].

In addition, a substantial proportion of the respondents (66.9%) agreed that customers who came to their pharmacy frequently accepted their product recommendations, affirming the role of community pharmacists as a trustworthy source of health-related advice. However, they may not be the go-to health professionals when women seek advice on managing PMS symptoms. In a prior study [11], pharmacists, when rated alongside other health professionals, were the least ‘sought after’ source of therapeutic recommendations for PMS. The study also showed that many of the participants preferred self-medicating or obtaining advice from family members and friends about managing PMS.

### 4.3. The Respondents’ Pattern of Pharmacological Recommendations

We found that the most frequently reported affective symptom to our respondents was irritability. Similar findings were reported in a prior study that involved female medical students at Universiti Malaysia Sabah [23]. Another study reported that confusion, depression, social withdrawal, anger, and anxiety were less common than irritability among high school students [2].

The most common somatic symptoms reported to our respondents were headache and abdominal bloating; two other studies recorded similar findings [2,33]. Indeed, the two symptoms have been noted to cause considerable distress in women with PMS and to frequently prompt them to seek treatment recommendations [23]. According to the ACOG’s diagnostic criteria for PMS, women must experience one or more of the somatic and affective symptoms during the five days before menses in each of the three previous menstrual cycles [34]. Taken together, the surveys of PMS symptoms have shown that PMS can manifest itself variably, and the management of PMS may benefit from a personalised approach.

Symptomatic treatment relieves a disease without addressing the underlying cause. We have shown that symptom-specific products (analgesics) were frequently recommended by the respondents to customers with PMS. A recommended drug regimen is taking ibuprofen, naproxen, or mefenamic acid during the luteal phase and stopping the drug after menses have begun [35]. Of the health supplements, evening primrose oil was the most preferred by the respondents. This shows that the product recommendations made by the respondents were based on sound evidence. Evening primrose oil can be an effective, alternative treatment for PMS, as it is rich in anti-inflammatory gamma linoleic acid and has been found to alleviate premenstrual breast pain in some women [36].

In choosing a symptom-targeting product, both the respondent’ and their customers preferred NSAIDs. This is consistent with the findings of a previous study, which showed that consumers with PMS often chose to use NSAIDs to relieve breast tenderness [11]. Another study also found that NSAIDs were among the non-prescription or OTC products most commonly used by Malaysian women to counter PMS symptoms, alongside vitamin supplements and health diets [12]. Two other studies highlighted that the drugs chiefly used for PMS were those having analgesic or anti-inflammatory effect [37,38]. There is a psychological need among women to reduce or prevent pain caused by PMS, and this often leads to self-medication [38]. Overall, health supplements were less frequently used by women with PMS, as those products are not sufficiently rapid-acting to bring about quick symptomatic relief. They must be taken for some time before their effect becomes apparent.

## 5. Conclusions

Our study is the first to explore the knowledge, attitudes, and practices of Malaysian community pharmacist in PMS management. We found that they had above-average levels of knowledge of and positive attitudes towards PMS management. At community pharmacies, this can lead to better treatment suggestions that are tailored to the robustness of the supporting evidence and the symptoms experienced by the customers.

Community pharmacists can take on a proactive role in the management of PMS, giving recommendations on rapid-acting non-prescription drugs, such as analgesics, for symptomatic relief. Besides, they can advise women with PMS on effective non-drug strategies such as lifestyle changes and the use of health supplements or natural products. Women with PMDD, a severe form of PMS that is unresponsive to non-prescription treatment, should be referred to gynaecologists. From the results of our survey, having taken courses on minor illnesses in women had positive effects on the respondents’ knowledge of PMS management. Therefore, we suggest that pharmacy schools may consider incorporating such courses into their core curricula.

A major limitation of our study is that it was conducted in only one of the federal territories of Malaysia, i.e., Kuala Lumpur. The findings may not represent the entire community pharmacy sector in Malaysia. However, Kuala Lumpur is the capital city of Malaysia with a high density of community pharmacies providing relatively advanced pharmaceutical care. The community pharmacists in the city are the likely forerunners effecting important changes to the pharmacy practice in Malaysia.

Another limitation of our study is that we used self-administered questionnaires to garner responses, and this could cause information bias. In future studies, a larger sample can be assembled to obtain more insight into the readiness of community pharmacists to help women in self-managing PMS and to determine the perceived barriers faced by community pharmacists in PMS management. In addition, a survey can be carried out to obtain public feedback on the possibility of establishing a specialised PMS management service that is provided by community pharmacists.

## Figures and Tables

**Table 1:** The demographic characteristics of the respondents (n = 181).

Demographic Characteristic	Frequency (n)	Percentage (%)
**Gender**		
Male	98	54.1
Female	83	45.9
**Age (years)**		
21–30	91	50.3
31–40	58	32.0
41–50	25	13.8
51–60	7	3.9
**Race**		
Malay	19	10.5
Chinese	150	82.9
Indian	10	5.5
Others	2	1.1
**Having graduated from a (first professional degree)**		
Local university	129	71.3
Overseas university	52	28.7
**Highest academic qualification**		
Bachelor	163	90.1
Master	16	8.8
PhD	1	0.6
Others	1	0.6
**Years as a fully registered pharmacist**		
1–5	93	51.4
6–10	35	19.3
11–15	19	10.5
16–20	16	8.8
21–25	14	7.7
26–30	4	2.2
**Years as a community pharmacist**		
1–5	118	65.2
6–10	26	14.4
11–15	14	7.7
16–20	17	9.4
21–25	4	2.2
26–30	2	1.1
**Location of the community pharmacy**		
Titiwangsa	11	6.1
Setiawangsa	14	7.7
Seputeh	27	14.9
Bandar Tun Razak	17	9.4
Wangsa Maju	15	8.3
Lembah Pantai	7	3.9
Bukit Bintang	16	8.8
Segambut	17	9.4
Batu	13	7.2
Cheras	29	16.0
Kepong	15	8.3
**Employment as a community pharmacist**		
Full time	158	87.3
Locum	23	12.7
**Having taken courses on managing minor illnesses in women at pharmacy school**		
Yes	115	63.5
No	66	36.5
**Where did you learn the most about managing PMS?**		
Pharmacy school	75	41.4
Practice experience	86	47.5
Continuing education	20	11.0
**Have you ever come across a customer with PMS?**		
Yes	181	100.0
No	0	0.0
**If YES, how often does this happen?**		
Daily	20	11.0
Weekly	68	37.6
Monthly	83	45.9
Yearly	10	5.5

**Table 2:** The respondents’ knowledge of premenstrual syndrome (PMS) and its management (n = 181).

Knowledge Items	Correct, n (%)	Incorrect or Unsure, n (%)
**Correct statements**		
PMS can be defined as a recurrent disorder that occurs every month in the luteal phase of the menstrual cycle and remits with the onset of menstruation.	161 (89.0)	20 (11.0)
The affective symptoms of PMS are depression, anger, irritability, anxiety, confusion, and social withdrawal.	167 (92.3)	14 (7.7)
The somatic symptoms of PMS are breast pain, abdominal bloating, headache, and swelling of extremities.	169 (93.4)	12 (6.6)
PMS can decrease work productivity.	172 (95.0)	9 (5.0)
PMS can interfere with sleep quality.	176 (97.2)	5 (2.8)
Ibuprofen can relieve PMS symptoms.	176 (97.2)	5 (2.8)
Spironolactone can relieve PMS symptoms.	88 (48.6)	93 (51.4)
Regular exercise can alleviate PMS symptoms.	143 (79.0)	38 (21.0)
**Incorrect statements**		
PMS is preceded by the appearance of the secondary sex characteristics and body changes that occur sometime between the ages of 9 and 16.	72 (39.8)	109 (60.2)
PMS is that point in time when permanent cessation of menstruation occurs following the loss of ovarian activity.	125 (69.1)	56 (30.9)
PMS can increase the quality of life.	165 (91.2)	16 (8.8)
Sertraline can relieve PMS symptoms.	57 (31.5)	124 (68.5)
Increased intake of caffeine can alleviate PMS symptoms.	69 (38.1)	112 (61.9)
Increased fibre and carbohydrate intake can alleviate PMS symptoms.	82 (45.3)	99 (54.7)

**Table 3:** The levels of the respondents’ knowledge of PMS (n = 181).

Knowledge Level	Frequency (n)	Percentage (%)
Low (<9 points)	42	23.2
Medium (9–11 points)	109	60.2
High (12–14 points)	30	16.6

**Table 4:** The relation between the respondents’ demographic profile and their knowledge of PMS and its management (n = 181).

Demographic Characteristics	Frequency (n)	Median Knowledge Score (IQR)	*p*-Value
**Gender**			
Male	84	10 (2)	0.484 ^a^
Female	97	10 (3)	
**Employment status as community pharmacist**			
Full time	158	10 (2)	0.122 ^a^
Locum	23	11 (2)	
**Years as a fully registered pharmacist**			
≤10 years	128	10 (3)	0.440 ^a^
>10 years	53	10 (2)	
**Years as a community pharmacist**			
≤10 years	144	10 (2)	0.491 ^a^
>10 years	37	10 (2)	
**Having taken courses on managing minor illnesses in women at pharmacy school**			
Yes	115	10 (2)	0.002 ^a,b^
No	66	9 (3)	

^a^ Mann–Whitney U test, ^b^ Statistically significant at *p* < 0.05.

**Table 5:** The respondents’ attitudes toward their role in PMS management (n = 181).

Attitude Items	Frequency (n)	Percentage (%)
**Pharmacists should know which medicine is used to relieve PMS.**		
Strongly disagree/disagree	2	1.1
Neutral	15	8.3
Agree/strongly agree	164	90.6
**Pharmacists should know the non-medical way to ease PMS.**		
Strongly disagree/disagree	4	2.2
Neutral	19	10.5
Agree/strongly agree	158	87.3
**Pharmacists should have sufficient time to provide self-care recommendations to patients with PMS.**		
Strongly disagree/disagree	2	1.1
Neutral	32	17.7
Agree/strongly agree	147	81.3
**Pharmacists should be actively involved in the selection of self-care medications for patients with PMS.**		
Strongly disagree/disagree	0	0.0
Neutral	31	17.1
Agree/strongly agree	150	82.8
**Pharmacists should provide patients with information on the use of medicine for PMS.**		
Strongly disagree/disagree	0	0.0
Neutral	6	3.3
Agree/strongly agree	175	96.6
**Pharmacists should recommend a PMS-relieving product based on the scientific evidence.**		
Strongly disagree/disagree	5	2.8
Neutral	17	9.4
Agree/strongly agree	159	87.9
**Pharmacists need to explain the doses and the ways of taking medicine to ease PMS.**		
Strongly disagree/disagree	2	1.1
Neutral	6	3.3
Agree/strongly agree	173	95.6
**Pharmacists should inform patients of the side effects of PMS-relieving products.**		
Strongly disagree/disagree	2	1.1
Neutral	15	8.3
Agree/strongly agree	164	90.6
**Pharmacists should always check that their patients understand the information they are provided about PMS-relieving products.**		
Strongly disagree/disagree	2	1.1
Neutral	15	8.3
Agree/strongly agree	164	90.6
**Pharmacists need to be ready to respond to inquiries from customers about PMS-relieving products.**		
Strongly disagree/disagree	2	1.1
Neutral	10	5.5
Agree/strongly agree	169	93.4
**Patients regularly accept my recommendations for PMS-relieving products.**		
Strongly disagree/disagree	3	1.7
Neutral	57	31.5
Agree/strongly agree	121	66.9
**I am willing to provide recommendations for managing PMS when given the opportunity.**		
Strongly disagree/disagree	2	1.1
Neutral	14	7.7
Agree/strongly agree	165	91.1

**Table 6:** Rating the respondents’ attitudes toward their role in PMS management (n = 181).

Level of Attitude	Frequency (n)	Percentage (%)
Negative (<36 points)	2	1.1
Neutral (36–47 points)	37	20.4
Positive (48–60 points)	142	78.5

**Table 7:** The relation between the respondents’ demographic characteristics and their attitudes toward their role in PMS management (n = 181).

Demographic Characteristic	Frequency (n)	Median Attitude Ccore (IQR)	*p*-Value
**Gender**			
Male	84	50.50 (8)	0.486 ^a^
Female	97	51 (9)	
**Position as community pharmacist**			
Full time	158	51 (9)	0.561 ^a^
Locum pharmacist	23	51 (10)	
**Years as a fully registered pharmacist**			
≤10 years	128	51 (9)	0.871 ^a^
>10 years	53	51 (9)	
**Years as a community pharmacist**			
≤10 years	144	52 (9)	0.137 ^a^
>10 years	37	49 (9)	
**Having taken courses on managing minor illnesses in women at pharmacy school**			
Yes	115	51 (9)	0.153 ^a^
No	66	50.50 (9)	

^a^ Mann–Whitney U test.

**Table 8:** PMS symptoms often or always reported to the respondents (n = 181).

Symptom	Frequency (n)	Percentage (%)
Headache	117	64.6
Irritability	113	62.4
Abdominal bloating	109	60.3
Anger	73	40.3
Breast pain	65	35.9
Anxiety	46	25.4
Swelling of extremities	45	24.8
Depression	31	17.1
Social withdrawal	25	13.8
Confusion	17	9.4

**Table 9:** Products often or always recommended by the respondents to their customers (n = 181).

Product	Frequency (n)	Percentage (%)
Ibuprofen	143	79.0
Mefenamic Acid	135	74.5
Naproxen	121	66.9
Evening Primrose Oil	114	62.9
Soy	75	41.5
Magnesium	61	33.7
Vitamin E	59	32.6
Calcium	50	27.6
Vitamin B6	30	16.6
St. John’s Wort	20	11.1
Chasteberry	12	6.7
Saffron	10	5.5

**Table 10:** Products often or always requested by the respondent’s customers (n = 181).

Practice Items	Frequency (n)	Percentage (%)
Mefenamic Acid	148	87.7
Ibuprofen	132	79.0
Naproxen	132	72.9
Soy	36	19.9
Evening Primrose Oil	36	19.9
Magnesium	31	17.1
Calcium	30	16.6
Vitamin E	24	13.2
Vitamin B6	17	9.4
St. John’s Wort	8	4.4
Chasteberry	6	3.3
Saffron	6	3.3

**Table 11:** Respondents’ recommendations for the non-pharmacological management of PMS (n = 181).

Non-Pharmacological Measure	Frequency (n)	Percentage (%)
Eating a healthy, balanced diet	137	75.7
Rest and sleep more	135	74.6
Doing more exercise	125	69.1
Consult the doctor if the symptoms persist	125	69.1
Avoid caffeine and alcohol	123	68.0
Avoid stressful situations	103	56.9
Use an antipyretic if there is a fever	85	47.0
Talk to your friends or colleagues	68	37.6
No advice	3	1.7

**Table 12:** The reasons that explained why the respondents would encourage their customers to self-manage PMS symptoms (n = 181).

Reasons	Frequency (n)	Percentage (%)
Ease of availability and convenience	148	81.8
Cost saving	102	56.4
Time saving	57	31.5
Illness too trivial for consultation	56	30.9
Privacy	44	24.3
To avoid crowd at outpatient department	14	7.7
Lack of trust in prescribing doctor	8	4.4

**Table 13:** The reasons that explained why the respondents would discourage their customers to self-manage PMS symptoms (n = 181).

Reasons	Frequency (n)	Percentage (%)
Risk of using a wrong medication	139	76.8
Risk of adverse effects	126	69.6
Risk of disease misdiagnosis	125	69.1
Risk of drug interactions	105	58.0
Risk of drug abuse and dependence	94	51.9

## Supplementary Materials

The following are available online at https://www.mdpi.com/1010-660X/56/4/181/s1, Questionnaire: Community Pharmacists’ Knowledge, Attitude, and Practice in Providing Self-Care Recommendations for the Management of Premenstrual Syndrome.
