# Supplementary material for: Community Pharmacists’ Knowledge, Attitude, and Practice in Providing Self-Care Recommendations for the Management of Premenstrual Syndrome
Source: Medicina (Kaunas). 2020 Apr 15;56(4):181. doi: 10.3390/medicina56040181 (PMC7230671; doi:10.3390/medicina56040181)
Supplement: Supplementary file 1 [file medicina-56-00181-s001.pdf]

Community Pharmacists' Knowledge, Attitude, and Practice in Providing Self-Care Recommendations for the Management of Premenstrual Syndrome

Section A: DEMOGRAPHIC INFORMATION

No.	Item		Options			
**1.**	**Gender**	:	☐ **Male**		☐ **Female**	
**2.**	**Age**	:	___________ years			
**3.**	**Race**	:	☐ **Malay**	☐ **Chinese**	☐ **Indian**	☐ **Others:______**
**4.**	**Graduated from (For Bachelor of Pharmacy)**		☐ **Local university/college**		☐ **Overseas university/college**	
**5.**	**Highest academic qualifications**	:	☐ **Degree**	☐ **Master**	☐ **PhD**	☐ **Others:______**
**6.**	**Years as a registered pharmacist**	:	___________ years			
**7.**	**Years as a community pharmacist**	:	___________ years			
8.	**Location of community pharmacy**	:	☐ **Titiwangsa**	☐ **Wangsa Maju**	☐ **Batu**	
			☐ **Setiawangsa**	☐ **Lembah Pantai**	☐ **Cheras**	
			☐ **Seputeh**	☐ **Bukit Bintang**	☐ **Kepong**	
			☐ **Bandar Tun Razak**	☐ **Segambut**		

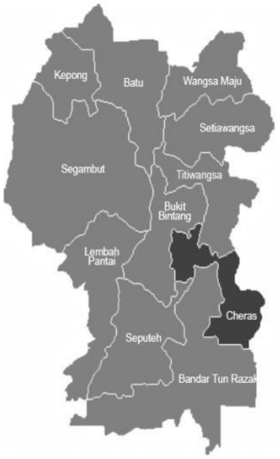

No.	Item		Options	
9.	**Position as community**	:	☐ **Full time pharmacist**	☐ **Part-time/locum pharmacist**
10.	**Did you take courses in pharmacy school regarding minor illnesses in women** i.e. Pre-menstrual syndrome		☐ **Yes**	☐ **No**

11.	**Where did you learn the most about managing Pre-menstrual syndrome (PMS):**	☐	**Pharmacy School**	☐	**Continuing Education**
		☐	**Practice Experience**	☐	**Others**<br>**Please specify: ________________**

Section B: KNOWLEDGE OF PRE-MENSTRUAL SYNDROME AND ITS MANAGEMENT

*Based on your **knowledge**, please **TICK** the appropriate answer from question **1 to 5.***

1A.	PMS is preceded by the appearance of the secondary sex characteristics and body changes that occur sometime between the ages of 9 and 16.	☐ True	☐ False	☐ I don't know
1B.	PMS can be defined as a recurrent disorder that occurs every month in the luteal phase of the menstrual cycle and remits with the onset of menstruation.	☐ True	☐ False	☐ I don't know
1C.	PMS is that point in time when permanent cessation of menstruation occurs following the loss of ovarian activity.	☐ True	☐ False	☐ I don't know
2A.	Affective symptoms of PMS are **depression, anger, irritability, anxiety, confusion** and **social withdrawal.**	☐ True	☐ False	☐ I don't know
2B.	Somatic symptoms of PMS are **breast pain, abdominal bloating, headache** and **swelling of extremities.**	☐ True	☐ False	☐ I don't know
3A.	PMS can increase quality of life.	☐ True	☐ False	☐ I don't know
3B.	PMS can decrease work productivity.	☐ True	☐ False	☐ I don't know
3C.	PMS can interfere with sleep quality.	☐ True	☐ False	☐ I don't know
4A.	Sertraline can relieve PMS symptoms.	☐ True	☐ False	☐ I don't know
4B.	Ibuprofen can relive PMS symptoms.	☐ True	☐ False	☐ I don't know
4C.	Spironolactone can relieve PMS symptoms.	☐ True	☐ False	☐ I don't know
5A.	Increased intake of caffeine can alleviate PMS symptom.	☐ True	☐ False	☐ I don't know

5B.	Increased fibre and carbohydrates intake can alleviate PMS symptoms.	☐ Yes	☐ No	☐ I don't know
5C.	Exercise regularly can alleviate PMS symptoms.	☐ Yes	☐ No	☐ I don't know

Section C: ATTITUDES OF COMMUNITY PHARMACISTS TOWARDS PMS MANAGEMENT

*Use the scale below to indicate how much you **AGREE** or **DISAGREE** with each of the following statements. **Circle** your answer in the space provided.*

1	Strongly Disagree	2	Disagree	3	Neutral
4	Agree	5	Strongly Agree		

1.	Pharmacists should know which medicine is used to relieve PMS.	1	2	3	4	5
2.	Pharmacist should know the non-medical way to ease PMS.	1	2	3	4	5
3.	Pharmacist should have sufficient time to provide self-care recommendation to patients with PMS.	1	2	3	4	5
4.	Pharmacist should actively involve in selection of self-care medication for the patients with PMS.	1	2	3	4	5
5.	Pharmacist should provide patients with information regarding the medicine use for PMS.	1	2	3	4	5
6.	Pharmacists should recommend a PMS-relieving product based on its scientific evidence.	1	2	3	4	5
7.	Pharmacists need to explain the doses and ways of taking medicines to ease the PMS.	1	2	3	4	5
8.	Pharmacists should inform patient of the side effect of the PMS relieving product.	1	2	3	4	5
9.	Pharmacists should always check that their patient understand the information they are provide about PMS-relieving product.	1	2	3	4	5
10.	Pharmacists need to be ready to respond to inquiries from customers about PMS-relieving product.	1	2	3	4	5
11.	Patients regularly accept my recommendations for PMS-relieving products.	1	2	3	4	5
12.	I am willing to provide recommendations for managing PMS when given the opportunity.	1	2	3	4	5

Section D: PRACTICE AND PATTERN OF PHARMACOLOGICAL RECOMMENDATION

Please ***TICK*** *in the space provided to indicate your answer for each of the following* ***questions 1 and 2****.*

1. Have you ever received a customer with PMS? ☐ Yes ☐ No

   *(If your answer is '****YES****', go to Question 2. If your answer is '****NO****', go to Question 4)*

2. If '**YES**', how often does this happen? ☐ Daily ☐ Weekly ☐ Monthly ☐ Yearly

Please rate your level of agreement for the following statements by ticking in the appropriate box from ***question 3 until 5****.*

3. Which of the following PMS **symptoms** is/are **often mentioned by customers** coming to your pharmacy?

**1**	**Never**	**2**	**Rarely**	**3**	**Sometime**
**4**	**Often**	**5**	**Always**	**6**	**Not Applicable**

	**1**	**2**	**3**	**4**	**5**	**6**
Breast Pain						
Anger						
Abdominal Bloating						
Irritability						
Headache						
Anxiety						
Swelling of Extremities						
Confusion						
Depression						
Social withdrawal						

4. For each of the products listed below, how often have you **recommended** them to your customers with PMS?

**1**	**Never**	**2**	**Rarely**	**3**	**Sometime**
**4**	**Often**	**5**	**Always**	**6**	**Not Available**

	**1**	**2**	**3**	**4**	**5**	**6**
Calcium						
Magnesium						
Chasteberry						
Vitamin B6						
Vitamin E						
Saffron						
St. John’s Wort						
Soy						
Evening Primrose Oil						
Ibuprofen						
Mefenamic Acid						
Naproxen						

5. For each product listed below, how often this product was **asked** by your customers?

	**1**	**2**	**3**	**4**	**5**	**6**
Calcium						
Magnesium						
Chasteberry						
Vitamin B6						
Vitamin E						
Saffron						
St. John’s Wort						
Soy						
Evening Primrose Oil						
Ibuprofen						
Mefenamic Acid						
Naproxen						

6. If there is any suggested product by you to your PMS customer(s), other than the above listed products, you may share it below:

....

Section E: PRACTICE OF NON-PHARMACOLOGICAL AND SELF-MEDICATIONS MEASURE

*Please **TICK** the appropriate answers for the questions **1 to 7.** You may choose **MORE THAN ONE ANSWERS.***

1. Which of the following represent(s) your advice to your customer(s) for the management of PMS?

- [ ] No advice
- [ ] Avoid stressful situation
- [ ] Doing more exercise
- [ ] Eating healthy balance diet
- [ ] Rest and sleep more
- [ ] Avoid caffeine and alcohol
- [ ] Use antipyretic if fever present
- [ ] Talk to your friend or colleagues
- [ ] Consult the doctor if the symptoms persist
- [ ] Others: Please specify: ____________________

2. What was (were) the reason(s) that you encourage your customers with PMS to practice self-medication?

- [ ] Illness too trivial for consultation
- [ ] Privacy
- [ ] Avoid crowd at OPD
- [ ] To save time
- [ ] Ease of availability and convenience
- [ ] Cost saving
- [ ] Lack of trust in prescribing doctor
- [ ] Others: Please specify: ____________________

3. What the reason(s) do you against your customer(s) with PMS to practice self-medication?

- [ ] Risk of drug abuse and dependence
- [ ] Risk of drug interactions
- [ ] Risk of disease misdiagnosis
- [ ] Consult a doctor
- [ ] Risk of using wrong medications
- [ ] Risk of adverse effects
- [ ] Others:
- [ ] Please specify: ______________________

4. What do you think about self-medication for PMS management?

- [ ] Good practice
- [ ] Acceptable practice
- [ ] Not acceptable practice
